# THE ASSOCIATION BETWEEN FRAILTY AND PERCEIVED FATIGABILITY IN THE LONG LIFE FAMILY STUDY

**DOI:** 10.1093/geroni/igac059.3089

**Published:** 2022-12-20

**Authors:** Benjamin Schumacher, Dustin Kehler, Alexander Kulminski, Stacy Andersen, Theresa Gmelin, Kaare Christensen, Mary Wojczynski, Nancy Glynn

**Affiliations:** University of Pittsburgh, Pittsburgh, Pennsylvania, United States; Dalhousie University, Halifax, Nova Scotia, Canada; Duke University, Durham, North Carolina, United States; Boston University School of Medicine, Boston, Massachusetts, United States; University of Pittsburgh, Pittsburgh, Pennsylvania, United States; Southern Denmark University, Odense, Syddanmark, Denmark; Washington University, Saint Louis, Missouri, United States; School of Public Health, University of Pittsburgh, Pittsburgh, Pennsylvania, United States

## Abstract

Higher levels of frailty, quantified by a frailty index (FI), may be linked to fatigue severity as tasks become more physically and mentally demanding. However, the association between frailty and fatigability—quantification of vulnerability to fatigue in relation to specific intensity and duration of activities—has not been assessed. Using cross-sectional data from the Long Life Family Study Visit 2 (2014–2017; n=2,524; mean age +/- standard deviation 71.4+/-11.2 years; 55% women; 99% White), we examined the association between a 79-item FI (ratio of number of health problems reported (numerator) out of the 79 (denominator); higher percentage=greater frailty) and perceived physical and mental fatigability using the Pittsburgh Fatigability Scale (PFS) (range 0–50; higher scores=greater fatigability). Mean+/-SD FI scores were 0.08+/-0.06 and mean+/-SD PFS Physical and Mental scores were 13.7+/-9.6 (39.5% more severe, >=15) and 7.9+/-8.9 (22.8% more severe, >=13), respectively. Both PFS subscale scores were higher for each 0.10 increment in FI. Mean PFS scores were 10.7 and 34.2 (Physical) and 5.7 and 28.8 (Mental) for FI scores of < 0.10 (non-frail) and ≥0.30 (moderate-severely frail), respectively. In mixed effects models, a 0.03 higher FI score (accepted clinically meaningful increase in FI) was associated with 1.9-point higher PFS Physical (95% confidence interval (CI) 1.7–2.1) and 1.7-point higher PFS Mental (95% CI 1.5–1.9) scores after accounting for family structure and adjusting for age, sex, field center, body mass index, smoking status, education, and marital status. Individuals with higher FI scores may benefit from targeted interventions to mitigate further poor health outcomes.

